# Mismatch between physicians and family members views on communications about patients with chronic incurable diseases receiving care in critical and intensive care settings in Georgia: a quantitative observational survey

**DOI:** 10.1186/s12904-016-0135-2

**Published:** 2016-07-22

**Authors:** Nana Chikhladze, Elene Janberidze, Mariam Velijanashvili, Nikoloz Chkhartishvili, Memed Jintcharadze, Julia Verne, Dimitri Kordzaia

**Affiliations:** Iv. Javakhishvili Tbilisi State University (TSU), Tbilisi, Georgia; Department of Gerontology and Palliative Care, of Al. Natishvili Institute of Morphology, TSU, Tbilisi, Georgia; Georgian National Association for Palliative Care, Tbilisi, Georgia; Infectious Diseases, AIDS and Clinical Immunology Research Center, Tbilisi, Georgia; Public Health England, Bristol, UK

**Keywords:** Observational survey, Critical/Intensive care physicians, Patients’ family members, Palliative care, Mismatch in views on communications

## Abstract

**Background:**

Physicians working in critical and intensive care settings encounter death of chronic incurable patients on a daily basis; however they have scant skills on how to communicate with the patients and their family members. The aim of the present survey is to examine communication of critical and intensive care physicians with patients’ family members receiving treatment due to chronic incurable diseases/conditions and to compare the views of families with physicians working in critical and intensive care settings.

**Methods:**

The survey was conducted in four cities of Georgia (Tbilisi, Kutaisi, Batumi and Telavi) in 2014. Physicians working in critical and intensive care settings and family members were asked to fill in separate questionnaires, covering various aspects of communication including patients’ prognosis, ways of death occurrence, treatment plans and religion. Participants ranked their responses on a scale ranging from “0” to “10”, where “0” represented “never” and “10”-“always”. After data collection, responses were recoded into three categories: 0–3 = never/rarely, 4–7 = somewhat and 8–10 = often/always. Differences were tested using Pearson’s chi-square or Fisher’s exact test as appropriate. P value of < 0.05 was considered as significant.

**Results:**

Sixty-five physicians and 59 patients’ family members participated in this cross-sectional study. Majority of their responses was statistically significantly different. Only one quarter (23.7 %) of family members of patients receiving medical aid in critical and intensive care settings were satisfied with the communication level. In contrast, 78.5 % of physicians considered their communication with families as positive (*p* < 0.0001).

**Conclusions:**

The survey revealed the mismatch between the views on communication of critical and intensive care settings physicians and family members of the patients with chronic incurable diseases receiving care in critical and intensive care settings. In order to provide the best care for chronic incurable patients and their family members, physicians working in critical and intensive care settings must have relevant clinical knowledge and ability to provide effective communication. Present results reflect important potential targets for educational interventions including critical and intensive care physicians training through online modules.

**Electronic supplementary material:**

The online version of this article (doi:10.1186/s12904-016-0135-2) contains supplementary material, which is available to authorized users.

## Background

Critical and Intensive Care (CIC) is the setting for the death of many cancer patients around the world, however the proportion of patients dying in acute care hospitals and in CIC settings vary [1]. Previously, concerns have been raised about the costs associated with admission to CIC for terminally ill cancer patients; at the same time, the issue related to the withholding and withdrawal of life prolonging treatment has always been challenging [2]. Therefore death in hospital is often contrary to patients’ wishes, many of whom would prefer to die at home [3–5]. Many patients with serious and life-threatening illness are admitted to CIC settings because the symptoms cannot be controlled at home or in the community setting [6, 7], however question about ‘futility’ of some types of care provided to terminally ill patients in CIC settings is being increasingly debated [8]. Although, many patients die in CIC settings, the purpose of being admitted is to attempt to prolong life. Expectations and hopes of the patients’ relatives are often raised. However, the CIC physicians may have limited training and resources to manage and respond well to patients who have palliative care needs, nor to fully respect these patients’ preferences and expectations [9, 10]. Despite encountering death on a daily basis physicians in CIC settings are not always trained in delivering bad news [11]. In addition, in Georgia, physicians do not feel comfortable to break bad news with patients or their relatives about poor prognosis or the futility of treatment [12].

In Georgia, after restoration of independence in April 9, 1991, there has not only been an improvement in the quality of medical care, but also significant change in medical values accompanying a return to Georgian traditions and culture. For example, in soviet times, when atheism dominated, death was considered to be something which medical technology should attempt to prevent at all costs, but not as the normal consequence of life accompanied by spiritual preparation for the patient and family. Restoration of independence returned Georgia to traditional cultural values, which included a strong influence of Orthodox Christianity. In the first years of independence, the “Law on Healthcare”, “Law on Patients’ Right” and “Law on Medical Activities” were approved by the Parliament of Georgia. In addition, Bioethical National Council has been created. Sharing of diagnosis with the patient and their family members became mandatory, therefore new regulations made doctors to re-consider death as natural part of end-of-life. This requires special preparation from medico-biological, as well as, spiritual points of view. Since 2007 palliative care has been recognised in Georgia as a sub-speciality for four specialties: “Internal Medicine”, “Surgery”, “Oncology” and “Reanimatology/Intensive Therapy”. Later, in 2014 the list of such specialties was increased up to 11 [13]. Implementation of educational/training courses in palliative care aimed to develop more holistic approach to the care of dying patients considering their physical, psychological social and spiritual needs. However, while the concepts of and training in palliative care are being implemented in Georgian medical field, some branches including CIC, still need more time to fully integrate these principles in their professional practice.

It is confirmed, that realization of patient’s imminent death leads to potential changes in management which includes clarifying of treatment goals, improving communication with families, spending more time with patients and ordering fewer laboratory tests [14]. However, a previous study conducted in 2009–2010 in two Georgian cities (Tbilisi and Batumi) showed that number of Georgian physicians, especially in CIC settings, due to their scant knowledge of palliative care, follow their traditional goals to “cure” the patient or “postpone death” [15]. The study showed that it may lead to neglecting or missing the opportunity to meet the more holistic needs of patients and their families. 29 from 2000 (14.5 %) of advanced cancer patients spent their last days and hours in CIC settings. Family members of more than half of these patients had different complains regarding the care provided to the patients and were dissatisfied with communication with medical staff.

Patients’ families play a significant role in demanding the transfer of advanced cancer patient to CIC setting. These demands are partially made because they are not aware that their relative is in a terminal condition and also because of continued hope of “delaying death” [15]. As a high proportion of the cancer patients who die in CIC have altered state of consciousness, families play an essential role in making decisions on their behalf [16]. This means that good communication between physicians and family members of advanced cancer patients in CIC settings is critical to ensure the best possible holistic care [14].

Studies conducted during the last decade in US and UK showed the importance of improvement and/or development of palliative care competencies in CIC settings [17, 18]. This implies the standards providing symptom control, pain relief and achievement of maximal physical comfort, as well as, consistency with humanistic goals, ethical and psychological support to patients and their families At the same time, implementation of palliative care approaches in CIC settings is related with difficulties of switching abruptly from curative approach to preparation for dying which itself may vary from several hours to several weeks [19]. To improve communication with family members is one of the important issues in this aspect [14].

The aim of this survey is to compare the views of family members of the patients receiving treatment due to chronic incurable diseases/conditions in CIC settings and CIC physicians—on various aspects of their essential communications regarding the patients (prognosis, ways of death occurrence, further treatment plan discussion, faith, spiritual attitudes and religiousness).

## Methods

### Study design, settings and participants

Cross-sectional study was conducted from February 2014 through July 2014. Convenience sampling approach was used and consecutive numbers of participants were enrolled over the study period.

The eligibility criteria: The physicians working in CIC settings in four cities of Georgia (Tbilisi, Kutaisi, Batumi and Telavi) with at least 4 years of work experience but with no specific training in palliative care.

Family members of adult patients (age ≥18 years) receiving medical care in the same hospitals of recruited physicians were invited to participate in the survey. Patients’ family members were chosen for the study as the contact with terminal incurable patients in CIC settings frequently is difficult or impossible due to patients have altered state of consciousness. The study exclusion criteria were patients’ family members with limited or no knowledge of Georgian language.

Physicians and patients’ family members were recruited independently, and physicians were not required to be the primary care provider of the recruited patient. Patients’ personal information (e.g. age, sex, diagnosis and date of death) was provided by patients’ family members based on standardized discharge/death certificate issued by the hospital (the data is available in Table 1).

**Table 1:** Characteristics of the patients whose family members participated in the study^a^ denotes the number of died patients

Age, Gender Diagnoses^a^	25–40		41–60		60–74		75–80		Sum
	Woman	Man	Woman	Man	Woman	Man	Woman	Man	
	2	3	6	7	11	9	9	12	
Breast Cancer, multiple metastases, multiorgan failure	1	–	3 (1)^a^	–	4 (1)	–	–	–	8 (2)
Pancreatic Head Cancer, Mechanical Jundice, Ascites.	–	–	1	1	–	1 (1)	1 (1)	1 (1)	5 (3)
Gallblader Duct Cancer, Cholangiostomic Obstruction	–	–	–	–	–	–	–	1 (1)	1 (1)
Liver Cancer, Hepatic Failure	–	–	–	1	–	2 (2)	–	–	3 (2)
Lung Cancer, Multiple Bone Metastases	–	1	1	–	–	1	–	1 (1)	4 (1)
Kidney Cancer, Multiple Bone Metastases	–	–	–	1	–	1	–	–	2
Mediastinal Hodgkin’s Lymphoma,Multiorgan Failure	–	–	–	–	1	–	1	–	2
Retroperitoneal Lymphoma, Multiorgan Failure	–	–	–	1	–	–	1 (1)	–	2 (1)
Diabetes, Osteoporoses, Femoral Head Fracture, Kidney Failure	–	–	1	–	–	–	2	–	3
Prostate Cancer, Ascites, Multiorganic Failure.	–	–	–	2	–	2 (1)	–	–	4 (1)
Acute Stroke, Cerebral Coma	–	–	–	–	1	–	–	2 (1)	3 (1)
Post Stroke Condition, Partial Intestinal Obstruction	1	1	–	–	1	2 (1)	1	1 (1)	7 (2)
Alzheimer’s Disease	–	–	–	–	1	–	–	1	2
Heart Failure, Dementia	–	–	–	–	–	–	1	1	2
Uterine Cancer, Multiple Metastases, Hydrothorax, Ascites	–	–	–	–	3	–	1	–	4
Esophageal Cancer, Gastrostoma, Dementia	–	–	–	–	–	–	–	1 (1)	1 (1)
Large Intestinal Cancer, Terminal Ileostoma,	–	–	–	–	–	–	1	2 (1)	3 (1)
Liver Ciroses, Esophageal Varicose Bleeding, Multiorganic Failure	–	1	–	1 (1)	–	–	–	1 (1)	3 (2)
Sum	5		13 (2)		20 (6)		21 (9)		59 (17)

^a^denotes the number of died patients

### Instrument

The two separate questionnaires with parallel questions for physicians and family members were designed by team of experts working in CIC settings, palliative care, psychology, and ethics. The questionnaire was covering various aspects of communication including, but not limiting to, patients prognosis, expected time of death, treatment plans and religion. Questionnaires were reviewed by field experts for content validity and was piloted in six volunteers not taking part in survey (three CIC physicians and three family members of patients with cancer died in CIC setting) to ensure that all participants understand the questions correctly. The list of questions used in the study is shown in Table 2. The questionnaires were self-administered in presence of one of the researchers who explained the instrument to participants. Participants were asked to rank their responses on a scale ranging from 0 to 10 where “0” represented “never” and “10”-“always”. But for questions Q1.9–Q2.9–“0” represented “very difficult” and “10”–“absolutely not difficult” and for questions Q1.10–Q2.10–“0” represented “fully Negative” and “10”-“fully positive”.The data presented in Table 2 was disintegrated into separate figures in order to show the results of interviewing for each question in more simplicated way (see Additional file 1: Figure S1 - S10).

**Table 2:** Descriptive statistics of the responses from the physicians working in Critical and Intensive Care (CIC) settings and patients’ family members

Responses Physicians/patients’ family members	Never/Rarely		Somewhat		Often/Always		Don’t know		Overall *P* value
	*n* (%)	*P* value	*n* (%)	*P* value	*n* (%)	*P* value	*n* (%)	*P* value	
Physicians Q1.1. Do you talk with the patient and/or family members about the details, related to the possible deterioration of the health condition of the patient?	1 (1.5)	0.003	8 (12.3)	0.004	56 (86.2)	<0.0001	0	0.48	<0.0001
Patients’ family members Q2.1 Did the doctor talk with you, as the family member, about the details, related to the possible deterioration of the health condition of the patient?	10 (16.9)		20 (33.9)		28 (47.5)		1 (1.7)		
Physicians Q1.2 Do you inform the patient and/or family members, about the exact time interval the patient might live?	12 (18.5)	0.17	24 (36.9)	0.88	26 (40.0)	0.61	3 (4.6)	0.25	0.26
Patients’ family members Q2.2 Did the doctor inform you, as the patient’s family member, about the exact time interval the patient might live?	17 (28.8)		21 (35.6)		21 (35.6)		0		
Physicians Q1.3 Do you discuss with the patient how his/her death may occur?	46 (70.8)	–	17 (26.2)	–	2 (3.1)	–	0	–	–
Patients’ family members	–	–	–	–	–	–	–		
Physicians Q1.4Do you discuss with the family members about how the patient’s death may occur?	10 (15.4)	<0.0001	29 (44.6)	0.66	26 (40.0)	0.002	0	–	0.0004
Patients’ family members Q2.4 Did the doctor discuss with you about how the patient’s death may occur?	26 (4.1)		24 (40.7)		9 (15.3)		0		
Physicians Q1.5 Did you discuss the further treatment strategy (e.g. CPR, use of breathing support devices,) with the patient and/or family members?	3 (4.6)	0.07	7 (10.8)	0.02	55 (84.6)	0.001	0 00o0	–	0.004
Patients’ family members Q2.5 Did the doctor discuss with you the further treatment strategy e.g. CPR, use of breathing support devices?	9 (15.3)		16 (27.1)		34 (57.6)		0		
Physicians Q1.6 Do you talk to the patient and/or family member about life-related topics associated and important to the patient?	7 (10.8)	<0.0001	33 (50.8)	0.002	23 (35.4)	0.10	2 (3.1)	0.81	<0.0001
Patients’ family members Q2.6 Did the doctor talk with you about life-related topics associated and important to the patient?	30 (50.8)		14 (23.7)		13 (22.0)		2 (3.4)		
Physicians Q1.7 Do you talk with the patient about his/her faith (religion)?	23 (40.0)	<0.0001	23 (40.0)	0.02	11(16.9)	0.05	0	–	<0.0001
Patients’ family members Q2.7 Did the doctor talk with about the Patient’s faith (religion)?	46 (78.0)		10 (16.9)		3 (5.1)		0		
Physicians Q1.8 Do you respect the spiritual attitudes and religiousness of the patient?	0 (0.0)	<0.0001	23 (35.4)	0.01	42 (64.6)	<0.0001	0 (0.0)	<0.0001	<0.0001
Patients’ family members Q2.8 Did the doctor show respect towards the patient’s spiritual attitudes and religiousness?	29 (49.2)		9 (15.3)		4 (6.8)		17 (28.8)		
	Very difficult		Somewhat		Not at all		Don’t know		
Physicians Q1.9 Do you find it difficult to talk with the patient and/or his family about the patient’s death?	11 (16.9)	0.05	39 (60.0)	0.40	15 (23.1)	0.02	0	–	0.0219
Patients’ family members Q2.9 Was it difficult for the doctor to talk with you about the patient’s death?	3 (5.1)		31 (52.5)		25 (42.4)		0		
	Negative		Somewhat		Positive		Don’t know		
Physicians Q1.10 How would you appraise your communication with the patient in general?	1 (15.0)	0.001	13 (20.0)	<0.0001	51 (78.5)	<0.0001	0	–	<0.0001
Patients’ family members Q2.10 How would you appraise doctors’ communication with you in general?	12 (20.3)		33 (55.9)		14 (23.7)		0		

Participants also had the option “do not know” for every question. Each participant had only one session for filling questionnaires.

### Statistical analysis

After data collection, responses to the questionnaires were recoded into three categories: 0–3 = never/rarely, 4–7 = somewhat and 8–10 = often/always. Answers on “do not know” were classified separately.

Standard, descriptive statistics were conducted to access variable distributions. Comparisons between physician and patients’ family members responses were tested in bivariate analysis for each individual questionnaire item. Differences were tested both for entire item and for each individual response category using Pearson’s chi-square or Fisher’s exact test as appropriate. Fisher’s exact test was used if the number of responses in any category to be tested was ≤5, otherwise preference was given to Pearson’s chi-square test [20]. Two-sided significance tests were used and *p* value of < 0.05 was considered as significant.

## Results

A total of 68 physicians working in CIC settings were approached, among them 65 (response rate 95.6 %) agreed and took part in the survey.. The age range of the physicians was 31–74, female−25, male−40. Professional experience of the physicians varied from 4 to 48 years.

Of 70 family members of patients with chronic incurable disease receiving care in CIC settings were approached and 59 (response rate 84.3 %) participated in the survey. Results of quantitative analysis for the responses on both questionnaires are presented in Table 2.

Comparison of physicians’ and patients’ family members” responses to paired questions showed substantial differences. Except for question Q1.2/Q2.2 on patient’s life expectancy, differences in responses to all other items were statistically significant. For example, the vast majority of physicians (86.2 %) reported that they often/always discussed the possible deterioration of a patient’s condition whereas the less than half of patients’ family members (47.5 %) reported often/always being given this information (*p* < 0.0001).

Seventy point eight percent of physicians reported that they have never/rarely discussed with the patient how death may occur (Q1.3), but 40 % reported that they often/always have this discussion with patients’ family members (Q1.4). However, only 15.3 % of patients’ family members confirmed such discussions (Q2.4) (*p* = 0.002).

The majority of physicians (84.6 %) reported to have discussions about future treatment plans, (e.g. use of cardiopulmonary rresuscitation or breathing support devices) as “often/always”, but the same assessment was reported from patients’ family members only in 57.4 %.

Half (50.8 %) of patients’ family members reported that physician never/rarely talked about life related topics associated and important to the patient, as compared to 10.8 % reported by physicians Q1.6/Q2.6 (*p* < 0.0001). As for the respect to patients’ spiritual attitudes and religiousness, 64.6 % of CIC physicians said that they often/always had demonstrated such respect, while only 6.8 % patients’ family members felt that the physicians did so (Q1.8/Q2.8) (*p* < 0.0001).

Only one quarter (24.8 %) of patients’ family members receiving medical aid in CIC settings were satisfied with the communication level (Q2.10). The reason for their dissatisfaction was sense of inadequate appreciation of their needs and requirments. In contrast, 78.5 % of CIC physicians considered their communication with patients’ family members as positive (Q1.10) (<0.0001).

## Discussion

This is the first survey conducted in CIC settings in Georgia, an example of the country where medical care is developing according to western standards [21], however communication skills still lag [12]. We examined the communication between physicians and family member of the patients dying from chronic incurable diseases in CIC settings. We wanted to demonstrate the possible mismatch between what physicians say and what families say about their communication. For almost every question the answers provided by physicians and patients’ family members significantly differ from each other. The exception is the assessment of communication regarding the prognosis, in particular, the length of time the patient was expected to live. For this question the physicians and family members gave a similar evaluation of the communication process: 55,4 % of physicians and 64,4 % of patients’ family members recognize that this question was addressed “never/rarely” or “somewhat”. It may reflect that the real difficulty lies in establishing precise prognosis, thus physicians may be cautious about communicating how long the patient can live. The significant contrast in responses to paired questions highlights the problems in communication between CIC physicians and patients’ family members in this aspect. Despite the fact, that 40,0 % of CIC physicians think, that they”often” discuss with patients’ family members how and in what conditions the patient could die, these assumptions are confirmed only by 15,3 % of patients’ family members (*p* < 0,002). The similar differences in responses are revealed for questions regarding the communication about further treatment plans: 84,6 % of CIC physicians think that they “often/always” co-ordinate treatment plan with patients’ family members, while only 57,6 % of relatives thinks that they do. Some of this difference may reflect the state of stress of the patients’ family members when they receive bad news about the prognosis of their loved ones in CIC settings. Acting as a surrogate decision maker is known to be a very stressful situation which can affect recalling of the information given [11]. Effective communication between critical care providers and surrogates, who are often family members, is a critical part of informed decision making regarding goals of care, including whether or not to pursue cardiopulmonary resuscitation in the intensive care unit [18]; End-of-life decisions for patients with surrogates usually are made at family conferences with participation of ethicists if needed [22]. Development of similar practice in Georgia would be highly appreciated.

The answer—“do not know” was rarely recorded except in the question regarding respect towards patient’s spiritual attitudes and religiousness: 17 patients’ family members (28,8 %) gave this answer, whereas none of the physicians did so. It was revealed that 40 % of CIC physicians think that they “somewhat” considered patients’ religious believes and faith. However, the response “somewhat”, perhaps means, that medical staff simply addressed these aspects with restraint and/or hesitation, keeping a sense of distance. This restraint and hesitation on non-medical matters is reflected in the doubts of patients’ family members regarding the physicians’ attitudes towards patients’/patients’ family members’ religious and spiritual beliefs. The analyses of questionnaires indicate that physicians and patients’ family members have significantly different attitudes towards consideration of such aspects during patient care. This probably does not contribute to development of confidence among communicating sides and may be considered as one of the main causes of patients’ family members dissatisfaction by communication with CIC physicians. It appears physicians have the appropriate attitudes, just their knowledge and skills need improvement [23, 24].

Three-quarters (76.9 %) of CIC physicians as well as 57,6 % of patients’ family members confirm that physicians struggle and cannot talk freely, when discussing patient death with family members (Q1.9/Q2.9). Despite this fact, 78,5 % of CIC physicians perceive communication with patients’ family members as positive.

It is revealed that CIC physicians talk about patient death more frequently with patients’ family members, than with the patients (*p* < 0,001). On the one hand, this reflects the fact that frequently it is difficult or impossible to communicate with the patients due to their altered consciousness. On the other hand, this fact might be explained by deficiency of relevant communication skills in “breaking bad news” related with the gap in professional education—on all levels of specialization [12].

Implementation of the recognized strategy—“Palliative Care for everyone who needs it”—necessarily requires that all medical professionals, who may be involved in the management of end-of-life patients, including CIC physicians, must be competent in basic knowledge and skills of palliative care [25–28].

However, adopted standards for palliative care require CIC physicians to ensure quality symptom control, pain relief and achievement of maximal physical comfort on the one hand, and consistency with humanistic goals on the other which is particularly important for patients and patients’ family members [19]. They find it difficult to switch abruptly from the hope to survive and cure to preparation for dying and care at the end-of-life. Such transformation of medical care represents one of the most challenging steps in CIC settings [18, 29–31].

In general, both patients and patients’ family members remain dissatisfied with the decision to quit the curative measures and continue only with palliative care. This is related with disappointments of their hopes [23, 32–34]. Poor communication skills of CIC physicians complicate understanding and collaboration among the CIC setting staff, patients and patients’ family members. The persistence of the tradition of withholding the “bad diagnosis”, as well as “stigma” and “taboo” associated with terminal illnesses in Georgia, further aggravates the problem [12].

CIC physicians have to deal with last days and hours of life exclusively in CIC settings, where the death is extremely common. Therefore, studies implemented during the last decade showed the absolute necessity for the development and improvement of palliative care service in CIC setting. palliative care in CIC may be implemented via two main models: a) “consultative model”, when palliative care is delivered by invited specialists (consultants), who communicate with the patient and their family, develop, negotiate and implement the care plan themselves or supervise the process and b) “integrative model”, when the care of the patient is delivered immediately by the staff of CIC setting on everyday basis. The second model is considered to be more cost-effective and adequate than the first one. In addition, it does not interfere with continuity of palliative care in specialized departments [12, 34–36].

Several indicators of good medical care at the end of life are identified: adequate management of pain and other symptoms, avoiding unnecessary and inexpedient prolongation of treatment, achieving sense of control, relieving burden to others and strengthening relationships with the loved ones. Effective communication with patient’s family and consideration of their needs and wishes is equally important [37–41].

Before 2004 there was no official recognition of palliative care in Georgia. The updated standards of palliative carehave been introduced since 2004 by the implementation of pilot educational programs leaded by foreign experts [42] and aiming to give basic knowledge to healthcare professionals, policymakers and medical students. Later these were followed by approval of amendments in four laws of Georgia (“Law on Health Care”, Law on Medical Activities”, “Law on Narcotic Drugs, Psychotropic Substances and Precursors” and “Law on Patients Right”) by Georgian Parliament, issuing decrees regulating palliative care and including “Palliative care and Pain Medicine” as a “subspecialty” in the list of medical specialties by Ministry of Health of Georgia in 2007 and 2008 [12].

In the light of these achievements, development of special training and qualification courses in palliative care for CIC physicians emerged as the urgent goal. In 2014 we (N. Chikhladze, M. Velijanashvili, D. Kordzaia) prepared the online-based learning course which was accredited by the Continued Education Council (Department) at Faculty of Medicine, TSU. The rationale for using an online course was the desire to allow CIC physicians to develop their knowledge without leaving their work place, which is particularly very important for CIC setting staff. Apart from the basic skills in palliative care including the peculiarities of communication with chronic incurable patients and their families regarding end-of-life, how the death may occur, faith and religion, etc., this course provides specific knowledge about palliative care in emergencies and specifically during the last 48 h of life, involving purely medical care as well as family support and ethical-religious aspects.

Restoring of mandatory system of continued medical education and professional development in Georgia from 2017 (this system was canceled in 2004) should support the successful implementation of this course.

There are some limitations in this study. The study was cross-sectional involving a convenient sample of patients’ family members and physicians working in CIC settings. Therefore, this may affect generalisability of the present findings. Small sample size should also be mentioned, however, it was the first study conducted in Georgia to evaluate issues related to communication between physicians and patient representatives. Our study generated important evidence providing the basis for development of special training and qualification course in palliative care for CIC physicians.

## Conclusions

The survey conducted in four cities of Georgia revealed the mismatch between the views of CIC physicians and family members of the patients with chronic incurable diseases receiving care in CIC settings - on communication topics covering possible deterioration of a patient’s condition, future treatment plan, how death may occur, respect to patients’ spiritual attitudes and religiousness. Only one quarter (23.7 %) of patients family members were satisfied with the communication level. The reason for their dissatisfaction was sense of inadequate appreciation of their needs and requirements. In contrast, 78.5 % of physicians considered their communication with patients’ family members as positive.

This mismatch indicates that in order to provide the best care for chronic incurable patients and their family members in a holistic way the physicians of CIC settings must have both relevant clinical knowledge and skills as well as the ability to provide effective communication with patients and their families.

The notable discordance in responses to similar questions as well as overall assessment of communication by physicians and patients’ family members reflects important potential targets for educational interventions including CIC physicians training through online modules.

## Abbreviations

CIC, critical and intensive care; C.N., Chikhladze Nana; C.N., Chkhartishvili Nikoloz; DGPMIM, Department of Gerontology and Palliative Medicine of Alexandre Natishvili Institute of Morphology; GNAPC, Georgian National association for Palliative Care; J.E., Janberidze Elene; J.M., Jintcharadze Memed; K.D., Kordzaia Dimitri; TSU, Iv. Javakhishvili Tbilisi State University; V.J., Verne Julia; V.M., Velijanashvili Mariam.

## Additional file

Additional file 1: Distribution of responses from physicians and patients’ family members to question 1.1 and 1.2. **Figure S2.** Distribution of responses from physicians and patients’ family members to question 1.2 and 2.2. **Figure S3.** Distribution of responses from physicians to question 1.3. **Figure S4.** Distribution of responses from physicians and patients’ family members to question 1.4 and 2.4. **Figure S5.** Distribution of responses from physicians and patients’ family members to question 1.5 and 2.5. **Figure S6.** Distribution of responses from physicians and patients’ family members to question 1.6 and 2.6. **Figure S7.** Distribution of responses from physicians and patients’ family members to question 1.7 and 2.7. **Figure S8.** Distribution of responses from physicians and patients’ family members to question 1.8 and 2.8. **Figure S9.** Distribution of responses from physicians and patients’ family members to question 1.9 and 2.9. **Figure S10.** Distribution of responses from physicians and patients’ family members to question 1.10 and 2.10. (ZIP 702 KB)
