# Supplementary material for: Mismatch between physicians and family members views on communications about patients with chronic incurable diseases receiving care in critical and intensive care settings in Georgia: a quantitative observational survey
Source: BMC Palliat Care. 2016 Jul 22;15:63. doi: 10.1186/s12904-016-0135-2 (PMC4957836; doi:10.1186/s12904-016-0135-2)
Supplement: Additional file 1: — Distribution of responses from physicians and patients’ family members to question 1.1 and 1.2. Figure S2. Distribution of responses from physicians and patients’ family members to question 1.2 and 2.2. Figure S3. Distribution of responses from physicians to question 1.3. Figure S4. Distribution of responses from physicians and patients’ family members to question 1.4 and 2.4. Figure S5. Distribution of responses from physicians and patients’ family members to question 1.5 and 2.5. Figure S6. Distribution of responses from physicians and patients’ family members to question 1.6 and 2.6. Figure S7. Distribution of responses from physicians and patients’ family members to question 1.7 and 2.7. Figure S8. Distribution of responses from physicians and patients’ family members to question 1.8 and 2.8. Figure S9. Distribution of responses from physicians and patients’ family members to question 1.9 and 2.9. Figure S10. Distribution of responses from physicians and patients’ family members to question 1.10 and 2.10. (ZIP 702 KB) [file 12904_2016_135_MOESM1_ESM.zip › 12904_2016_135_MOESM5_ESM.pdf]

Figure 5. Distribution of responses from physicians and patients' family members to question 1.5 and 2.5

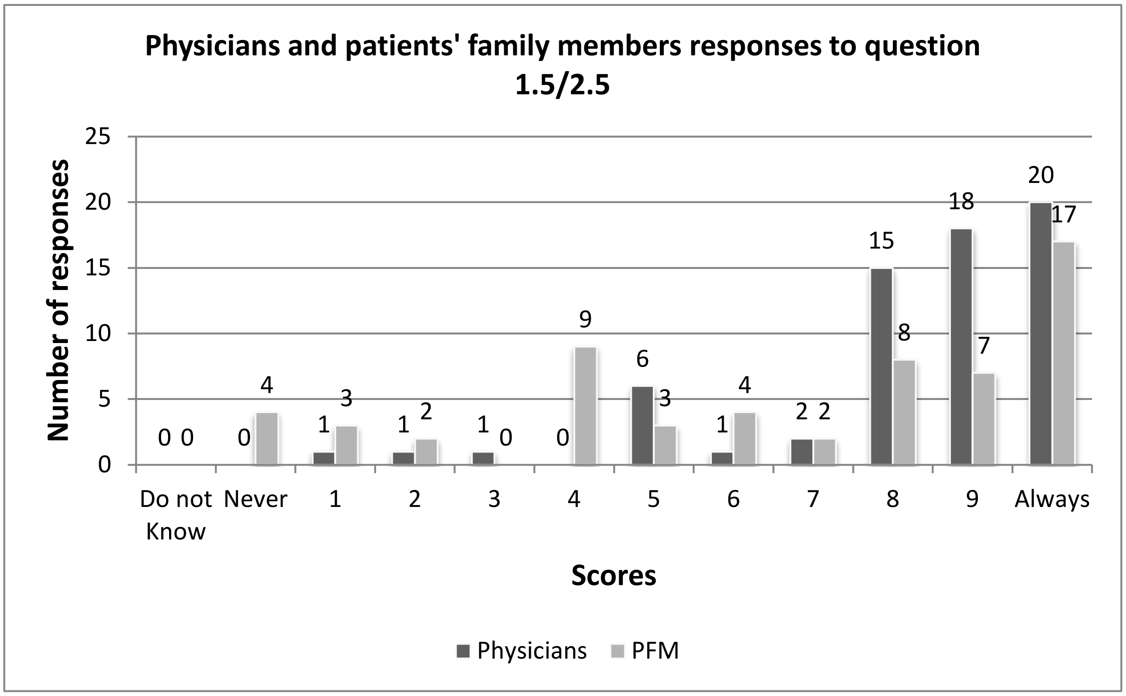

Black bars represent the distribution of physicians' responses, grey bars – responses of patients' family members. Numbering of figure indicates the question number in questionnaire.
